# Detection and quantification of microplastics in cerumen

**DOI:** 10.55730/1300-0144.6043

**Published:** 2025-06-29

**Authors:** Bengi ARSLAN, Yüce İSLAMOĞLU, Ali Sami BERÇİN, Selen AKBULUT, Mehmet MELİKOĞLU

**Affiliations:** 1 Clinic of Otorhinolaryngology, Ankara Bilkent City Hospital, Ankara, Turkiye; 2 Department of Otorhinolaryngology, Faculty of Medicine, Health Sciences University, Ankara, Turkiye; 3 Department of Otorhinolaryngology, Faculty of Medicine, Ankara Yıldırım Beyazıt University, Ankara, Turkiye; 4 Department of Food Technology, Üsküdar University, İstanbul, Turkiye; 5 Department of Chemical Engineering, Gebze Technical University, Kocaeli, Turkiye

**Keywords:** Microplastics, cerumen, human exposure, biomonitoring, excretion pathway

## Abstract

**Background/aim:**

Microplastics (MPs) are a growing concern due to their pervasive environmental presence and their potential impact on environmental and human exposure. Given evidence of systemic MP presence in human tissues and fluids, and the ear canal’s environmental exposure, this novel study aimed to identify these particles in human cerumen. Cerumen was collected from the proximal ear canal to minimize potential airborne contamination.

**Materials and methods:**

Cerumen samples were prospectively collected from 12 adult patients (23 patient-derived samples) and three control water samples, following ethical approval and consent. Samples were extracted using sterile instruments, stored in glass, diluted, and filtered through 0.22 μm cellulosic membranes. Microplastics were identified and measured morphologically using a 10× laboratory microscope with digital image processing; however, chemical confirmation of polymer type was beyond the scope of this initial study. Descriptive statistics were employed for analysis.

**Results:**

Among the 12 adult patients (23 patient-derived samples), microplastic particles were detected in 10 (83.3%, patient level detection rate). A cumulative total of 31 individual MPs were identified across the 23 patient-derived cerumen samples analyzed (comprising 29 detected and two instances of zero particle detection), ranging significantly in size from 16 μm to 930 μm and displaying various colors. Water control samples contained seven particles (3–46 μm), showing a clear size disparity from cerumen-borne particles.

**Conclusion:**

This study provides solid evidence of microplastic presence in human cerumen, suggesting a novel potential route of elimination from the human body. The high detection rate among patients and distinct characteristics of cerumen-borne MPs compared to controls imply genuine human accumulation rather than procedural contamination. Cerumen highlights a promising potential noninvasive bioindicator for assessing microplastic exposure. Further research in larger populations is essential to confirm these findings, elucidate mechanisms, identify polymer types, and explore potential health implications.

## 1. Introduction

Today, environmental pollution has become a significant public health problem due to its serious consequences. Plastic wastes are a major contributor to this issue [1]. The resulting small plastic pieces, known as microplastics (MPs), pose not only an environmental threat but also potential health concerns [2].

The term microplastic entered the scientific literature in 2004, referring to plastic particles smaller than 5 mm and insoluble in water [3]. Research has increasingly revealed the pervasive presence of microplastics in our daily lives, with detections in seafood, sea salt, and even drinking water [4,5]. Human studies have further identified microplastics in various tissues and fluids, including the intestine, placenta, lung, kidney, liver, blood, muscle, feces, breast milk, and urine [6–9]. These findings underscore the significant impact of plastic-related pollution and microplastics on our environment and raise questions about their potential effects on human well-being [10–12]. Given the ubiquitous presence of microplastics in the environment, including in the air we breathe, it is plausible that these particles could come into contact with and be trapped within bodily secretions like cerumen, which lines the directly exposed external auditory canal. Understanding the potential for microplastic accumulation in easily accessible bodily substances like cerumen could provide valuable insights into the extent of human exposure through environmental pathways. Consequently, the aim of this novel study was to investigate the presence of microplastics in human cerumen, specifically focusing on samples collected from the proximal ear canal to minimize potential contamination from external airborne sources.

## 2. Materials and methods

This prospective study was conducted following approval from the Ankara Bilkent City Hospital Clinical Research Ethics Committee (Ankara, Türkiye; E1-23–3235). Written informed consent was obtained from all patients prior to enrolment. For this investigation into the presence of microplastics in human cerumen, cerumen samples were collected from a total of 12 adult patients. A total of 23 patient-derived cerumen samples were analyzed, which, combined with the control samples, provided a robust initial group for comprehensive characterization, driven by the novel nature of this research question in the existing literature. However, it is important to note that this represents a pilot study, and thus future research with larger cohorts is essential for broader generalizability.

The inclusion criteria for participation were: age between 18 and 60 years, presence of cerumen in at least one external auditory canal, no current use of any treatment for cerumen impaction, and provision of informed consent to participate in the study. Exclusion criteria included: presence of tympanic membrane perforation, current or recent use of topical medications (e.g., ear drops) in the external auditory canal, regular use of hearing aids, headphones, or earplugs (defined as daily use exceeding 4 h), active infection of the external auditory canal, occupational history involving significant exposure to industrial dust or plastic manufacturing environments, and occurrence of bleeding during the cerumen sampling procedure.

### 2.1. Sample collection

Cerumen samples were carefully extracted from the external auditory canal of eligible patients using a sterile, single-use metal speculum (KARL STORZ SE & Co., Tuttlingen, Germany) and a sterile, single-use ear metal curette (KARL STORZ SE & Co., Tuttlingen, Germany), under direct visualization using an examination microscope (model: Leica M525 F50; Leica Microsystems, Wetzlar, Germany). To minimize potential contamination, the proximal portion of the cerumen, which had minimal direct exposure to the external environment, was collected and immediately transferred into precleaned borosilicate glass tubes (LEPUS Kimya Sanayi ve Ticaret Ltd. Şti., Tekirdağ, Türkiye). The opening of each tube was promptly sealed with a nonplastic cork stopper (LEPUS Kimya Sanayi ve Ticaret Ltd. Şti., Tekirdağ, Türkiye).

### 2.2. Laboratory analysis

The collected cerumen samples were processed and analyzed for microplastic content at the Food Engineering Laboratories of Yıldız Technical University (İstanbul, Türkiye) and Üsküdar University (İstanbul, Türkiye).

#### 2.2.1. Glassware preparation

All glassware used throughout the analytical process was meticulously rinsed three times with ultrapure water obtained from an ultrapure water purification system (Option-Q 7BP; ELGA, High Wycombe, UK) to eliminate potential background contamination.

#### 2.2.2. Sample processing

The initial volume or mass of each cerumen sample was recorded prior to dilution. To facilitate uniform sampling, each cerumen sample was first liquefied by gentle heating at 37 °C for 15 min. A 5 mL aliquot of the liquefied cerumen sample was then diluted with 20 mL of ultrapure water (1:4 dilution).

#### 2.2.3. Filtration of water control samples

To monitor for potential contamination from the ultrapure water used in the analysis, three 20 mL aliquots of the ultrapure water from the same batch used for sample dilution were filtered through 0.22 μm pore size cellulosic membrane filters (Filter Bio, Nantong, China). These control filters were processed and examined alongside the cerumen samples, providing an initial assessment of laboratory contamination, though a larger number of controls would enhance statistical confidence in contamination monitoring.

#### 2.2.4. Filtration of cerumen samples

The diluted cerumen samples were individually filtered through 0.22 μm pore size sterile cellulosic membrane filters (Filter Bio, Nantong, China) using a sterile glass filtration apparatus (Isolab, Eschau, Germany). Following filtration, the filters containing the retained particulate matter were carefully transferred to sterile glass petri dishes and allowed to air dry in a covered environment at room temperature to prevent airborne contamination. To minimize environmental contamination during analysis, all experimental procedures were performed within a HEPA-filtered chamber (S2020 1.2; Thermo Scientific, Waltham, MA, USA). Personnel wore cotton lab coats and nitrile gloves, and the chamber was thoroughly cleaned with 70% ethyl alcohol prior to the commencement of the study.

#### 2.2.5. Microplastic identification and characterization

The dried filters were examined under a camera light microscope (CKX41; Olympus Corp., Tokyo, Japan) equipped with a digital image processing system using a 10× objective lens. Microplastic particles were identified based on established morphological criteria, including consistent color, regular shape (e.g., fibers, fragments, films), distinct boundaries, and a lack of cellular or organic structures. Images of all identified microplastic particles were captured using the digital imaging system. The size (maximum Feret diameter) of each identified microplastic particle was measured using the image analysis software integrated with the microscope system (CKX41; Olympus Corp., Tokyo, Japan). It is important to note that identification relied solely on morphological characteristics, as advanced chemical characterization techniques (e.g., Fourier-transform infrared spectroscopy (FTIR) or Raman spectroscopy) for polymer confirmation were not performed in this study.

### 2.3. Statistical analysis

A total of 26 samples were analyzed in this investigation, comprising 23 patient-derived cerumen samples and three control water samples. Given the novel and exploratory nature of this pilot study, along with the relatively small sample size of individual patients (n = 12) and control samples (n = 3), the primary statistical approach focused on descriptive analysis. Frequencies and percentages of microplastic particle occurrence, along with measures of size range and distribution, were rigorously calculated using Microsoft Excel 2016 (Microsoft Corp., Redmond, WA, USA) to provide a thorough characterization of the observed data. Formal inferential statistical comparisons (e.g., t-tests, Mann–Whitney U tests) between cerumen and control samples were not performed due to the study’s pilot design and the insufficient sample size to achieve adequate statistical power for such hypothesis testing. This descriptive approach was deemed appropriate for precisely detailing the observed microplastic presence and identifying clear patterns and distinctions within the analyzed samples, laying the groundwork for future hypothesis-driven research.

## 3. Results

The experimental results are given in Tables 1–3 and summarized in the text below.

### 3.1. Characteristics of microplastics in cerumen samples

Microplastics were detected in 10 out of the 12 adult patient cerumen samples examined, yielding a detection rate of approximately 83.3% (patient level detection rate). In total, 31 individual microplastic particles were identified in cerumen samples collected from 12 adult patients; this count includes 29 particles detected in 10 patients and two instances of zero particle detection, representing the complete patient cohort. The size of these microplastics varied significantly, ranging from 16 μm to 930 μm. The colors observed in the cerumen microplastics included blue, green, red, black, navy blue, purple, dark blue, and transparent.

### 3.2. Comparative analysis of cerumen and control sample microplastics

Microplastics were detected in all three control water samples (100% detection rate), with a cumulative count of seven particles. These control particles exhibited a narrow size range of 3 μm to 46 μm. In contrast, patient-derived cerumen samples yielded a much higher cumulative total of 31 microplastic particles in cerumen samples collected from the 12 adult patients, despite two patient-derived samples showing no detection. Microplastics isolated from cerumen displayed a significantly broader and generally much larger size distribution, ranging from 16 μm to 930 μm. The smallest particle sizes observed in the controls (3–10 μm) were not found in the cerumen samples. Regarding color, control samples displayed five unique colors (black, red, green, transparent, blue-transparent), while patient-derived cerumen samples collectively exhibited eight unique colors (blue, green, red, black, navy blue, purple, dark blue, transparent).

### 3.3. Findings from individual samples

An examination of individual patient-derived cerumen samples revealed varied microplastic characteristics across the cohort. The first patient contained seven microplastic particles ranging from 16 μm to 383 μm, identified by their blue, green, red, and black colors. The second patient’s sample yielded three particles, larger in size at 370 μm to 646 μm, and colored navy blue and black. The third patient also had three particles, which were notably large, between 351 μm and 680 μm, and were navy blue and black. In contrast, the fourth patient’s sample showed no detectable microplastics. Moving to the subsequent samples, the fifth patient’s cerumen contained three particles ranging from 20 μm to 550 μm, colored black, blue, and purple. The sixth patient had a single, very large black particle measuring 930 μm. The seventh patient’s sample included two blue microplastics, sized 375 μm and 647 μm. The eighth patient presented with three particles, ranging from 59 μm to 677 μm, colored dark blue and black. The ninth patient’s sample contained two transparent particles, 42 μm and 75 μm in size. The tenth patient’s cerumen showed three particles between 405 μm and 770 μm, which were black, blue, and purple. Lastly, the eleventh patient had two transparent particles measuring 237 μm and 817 μm, while the twelfth patient, like the fourth, had no microplastics detected.

### 3.4. Summary of quantitative results

Microplastics were detected in all three control samples (100% detection rate), accounting for a total of seven particles. In contrast, cerumen samples collected from 10 out of 12 adult patients (83.3%, patient level detection rate) contained microplastics. A cumulative total of 31 microplastic particles were identified in cerumen samples collected from 12 adult patients; this count includes the 29 particles detected in 10 patients, along with two instances of zero particle detection, which are included to represent the complete patient cohort and ensure comprehensive representation. This represents a nearly 4.5-fold higher particle count in patient-derived samples compared to controls. Particle size distribution for control samples was exclusively within a narrow range of 3 μm to 46 μm. Conversely, microplastics in cerumen samples spanned a significantly broader and generally larger range, from 16 μm to 930 μm, with a complete absence of the smallest particle sizes (e.g., 3–10 μm) found in the controls. In terms of color, control samples displayed five unique colors (black, red, green, transparent, blue-transparent), while patient-derived cerumen samples collectively exhibited eight unique colors (blue, green, red, black, navy blue, purple, dark blue, transparent).

### 3.5. Microscopic images

**Figure 1:**
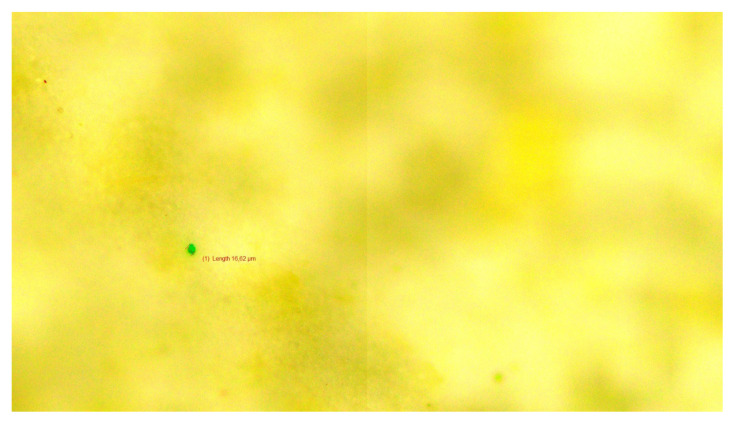
Image of the green microplastic particle detected in cerumen from the first experimental set.

**Figure 2:**
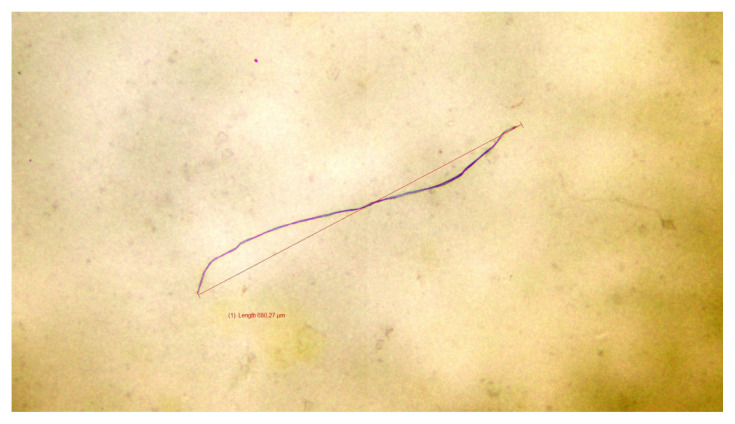
Image of the blue microplastic particle detected in cerumen from the second experimental set.

**Figure 3:**
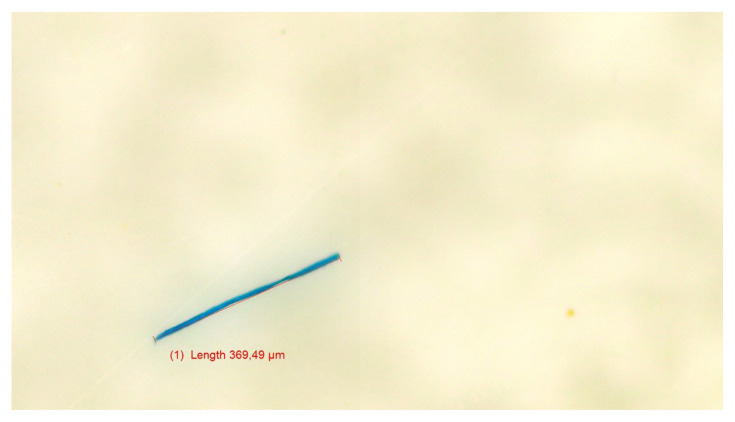
Image of the purple microplastic particle detected in cerumen from the third experimental set.

Figures 1 to 3 present representative microscopic images illustrating the morphology and diverse colors of the microplastic particles identified in cerumen samples from three sets of experiments.

## 4. Discussion

Cerumen, secreted within the external auditory canal, comprises a variety of substances and has a demonstrated utility in detecting biological markers, including viral presence [13–15]. Building upon the growing evidence of systemic microplastic presence in human fluids and tissues [16], this study hypothesized that these particles could be secreted into cerumen via the glands of the external auditory canal. Our findings support this hypothesis, demonstrating the presence of microplastics in a significant proportion of the analyzed cerumen samples and thereby suggesting cerumen’s potential utility as a noninvasive bioindicator for exposure assessment.

This research provides compelling evidence for the presence of microplastic particles within human cerumen, highlighting a novel and readily accessible biological matrix for assessing human microplastic exposure. Our findings demonstrate a high detection rate of microplastics in cerumen samples (83.3% of 12 adult patients), with a cumulative total of 31 particles identified across the patient cohort (comprising 29 individual particles detected from 10 patients, along with two instances of zero particle detection). This prevalence underscores the pervasive nature of microplastic contamination in the environment and its subsequent interaction with the human body.

The size of microplastics in cerumen varied significantly, ranging from 16 μm to 930 μm. This wide range indicates a diverse array of particle sizes accumulating within the cerumen. Similarly, the broad spectrum of colors observed, including blue, green, red, black, navy blue, purple, dark blue, and transparent, suggests multiple potential sources of microplastic exposure from the patients’ environments. The presence of specific colors such as navy blue, purple, and dark blue, which were not consistently found in the control samples, further hints at distinct sources of cerumen-bound microplastics.

A critical aspect of our investigation involved a rigorous comparison of microplastic characteristics in patient cerumen samples against those found in procedural water controls. While microplastics were universally detected in all three control samples, the cumulative particle count in these controls was notably lower (seven particles) compared to patient cerumen samples (31 particles), representing a nearly 4.5-fold higher particle count in patient samples. More significantly, a striking differentiation was observed in particle size distribution. Microplastics in control samples consistently exhibited a narrow size range (3 μm to 46 μm), typical of airborne or waterborne contaminants often associated with laboratory environments or collection procedures. In stark contrast, microplastics isolated from cerumen displayed a significantly broader and generally much larger size range (16 μm to 930 μm). This substantial size disparity, coupled with the complete absence of the smallest particle sizes (e.g., 3–10 μm) found in controls from the cerumen samples, strongly suggests that the microplastics detected in human cerumen are largely distinct from, and not primarily attributable to, contamination originating from the water used during the collection process. This finding is crucial as it indicates the cerumen microplastics likely represent genuine human exposure and accumulation.

Furthermore, the analysis of particle colors revealed a greater diversity in cerumen samples compared to controls. While some common colors (black, red, transparent) were observed in both, cerumen samples collectively exhibited a wider palette including blue, green, navy blue, purple, and dark blue, with certain hues not consistently present in the control batches. This varied color profile further supports the hypothesis that the microplastics in cerumen likely originate from multiple and diverse environmental or internal sources, reflecting the complex pathways of human microplastic exposure. The absence of detectable particles in two patient samples (the fourth and twelfth patients) is also an important finding, indicating variability in individual exposure, accumulation, or elimination, and highlights the need for further research into individual-level factors.

The detection of microplastics in cerumen suggests a potential pathway for the body to eliminate these foreign materials. This, coupled with the observed larger particle sizes in cerumen compared to water controls, implies human-based secretion, further supporting the utility of cerumen as a noninvasive bioindicator of microplastic exposure.

### 4.1. Limitations

While this study provides compelling initial evidence, a primary limitation is the relatively small sample size of 12 adult patients. Although this cohort allowed for a comprehensive preliminary characterization of microplastic presence in cerumen, these findings may not be broadly generalizable to the wider population. Consequently, the observed detection rate and particle characteristics should be interpreted with caution. This limitation underscores the need for subsequent research involving larger and more diverse human cohorts to validate these initial findings and enhance their statistical power and generalizability. Additionally, a key limitation lies in the relatively small number of water control samples analyzed (n = 3). While these controls were crucial for identifying baseline contamination from the ultrapure water and demonstrating a clear distinction from cerumen-borne particles, a larger and more varied set of control samples (e.g., air blanks, reagent blanks, field blanks) would have provided a more robust and statistically comprehensive assessment of potential laboratory and procedural contamination. This study also focused on descriptive characteristics (size, count, color) and did not include advanced analytical techniques such as FTIR or Raman spectroscopy to definitively identify the polymer types of the detected microplastics. This constitutes a significant limitation, as morphological identification alone cannot conclusively confirm a particle’s plastic composition or differentiate between various polymer types. Such chemical characterization would provide more definitive insights into their origins and allow for a more robust discussion of potential clinical implications and health risks. Furthermore, a key limitation lies in the current understanding of the precise mechanisms by which microplastics accumulate in and are eliminated via cerumen.

### 4.2. Future research

Building on these significant findings, subsequent studies should aim for larger and more diverse human cohorts to confirm these results and investigate potential correlations with environmental exposure, lifestyle factors, and critically, potential health outcomes. Such future studies, with adequately powered sample sizes, would enable the application of appropriate inferential statistical analyses to formally assess the significance of differences in microplastic characteristics between cerumen and various control groups, or across different patient demographics. Dedicated investigations are also warranted to elucidate the precise mechanisms of microplastic transport, accumulation, and elimination within the human body, particularly how these particles are delivered to and excreted via cerumen. The implications regarding the potential for cerumen as a noninvasive bioindicator and the pathways of microplastic elimination warrant further dedicated investigation, with a particular focus on understanding the clinical significance of microplastic accumulation in this matrix.

## 5. Conclusion

This original study provides solid evidence of the presence of microplastic in human cerumen, detected in a significant majority (83.3%, patient level detection rate) of the 12 adult patients from whom samples were collected. Across a total of 26 analyzed samples, including 23 patient-derived cerumen samples and three control water samples, a cumulative total of 31 microplastic particles were identified. This total comprises 29 individual particles detected in 10 patients, along with two instances of zero particle detection in the remaining two patients, reflecting the complete cohort and ensuring comprehensive statistical representation. These particles exhibited markedly larger sizes (16–930 μm) and a broader diversity of colors compared to microplastics found in procedural water controls (3–46 μm). This striking disparity in characteristics strongly suggests that cerumen-borne microplastics are not merely a result of external contamination during collection, but rather reflect genuine human exposure and a potential internal accumulation and elimination pathway. The detection of these ubiquitous environmental contaminants in a readily accessible human secretion underscores the pervasive nature of human microplastic exposure and their interaction with the human body. Building on the robust findings from this preliminary study, future research should prioritize confirming these observations in larger and more diverse cohorts, elucidating the precise mechanisms of accumulation and elimination, and identifying the polymer types of the detected microplastics. Ultimately, comprehensive studies are essential to fully assess the potential of cerumen as a noninvasive bioindicator for microplastic exposure and to cautiously investigate any potential health implications and inform public health strategies.

## Figures and Tables

**Table 1:** Results of laboratory microscope examination with digital image processing from the first experimental set.

Sample	Patient no.	Microplastic count	Size	Color
Control water sample 1	N/A	2 particles	39 μm and 46 μm	Black, red
Samples 1 and 2 were combined, as cerumen was present in both external auditory canals of the same patient	1	7 particles	16–383 μm	Blue, green, red, black
Samples 3 and 4 were combined, as cerumen was present in both external auditory canals of the same patient	2	3 particles	370–646 μm	Navy blue, black
**Total number of samples processed in this set: 4**	**Total number of patients: 2**	**Total microplastic count: 10 (Water control samples are not included)**	**Microplastic particle size range: 16–646 μm (Water control samples are not included)**	**Colors of the microplastics: blue, green, red, black, gray, navy blue**

**Table 2:** Results of laboratory microscope examination with digital image processing from the second experimental set.

Sample	Patient no.	Microplastic count	Size	Color
Control water sample 2	N/A	3	3 μm, 9 μm, and 10 μm	Red, green, transparent
Samples 6 and 7 were combined, as cerumen was present in both external auditory canals of the same patient	3	3	351–680 μm	Blue, black
Sample 8 (cerumen was present in only one external auditory canal of the patient)	4	Not detected	Not detected	Not detected
**Total number of samples processed in this set: 3**	**Total number of patients: 2**	**Total microplastic count: 3 (Water control samples are not included)**	**Microplastic particle size range: 351–680 μm (Water control samples are not included)**	**Colors of the microplastics: blue, black**

**Table 3:** Results of laboratory microscope examination with digital image processing from the third experiment set.

Sample	Patient no.	Microplastic count	Size	Color
Control water sample 3	N/A	2	39 μm and 46 μm	Blue-transparent
Samples 9 and 10 were combined, as cerumen was present in both external auditory canals of the same patient	5	3	20–550 μm	Black, blue, purple
Samples 11 and 12 were combined, as cerumen was present in both external auditory canals of the same patient	6	1	930 μm	Black
Samples 13 and 14 were combined, as cerumen was present in both external auditory canals of the same patient	7	2	375 μm and 647 μm	Blue
Samples 15 and 16 were combined, as cerumen was present in both external auditory canals of the same patient	8	3	59–677 μm	Dark blue, black
Samples 17 and 18 were combined, as cerumen was present in both external auditory canals of the same patient	9	2	42 μm and 73 μm	Transparent
Samples 19 and 20 were combined, as cerumen was present in both external auditory canals of the same patient	10	3	405–770 μm	Black, blue, purple
Samples 21 and 22 were combined, as cerumen was present in both external auditory canals of the same patient	11	2	297 μm and 817 μm	Transparent
Sample 23 (cerumen was present in only one external auditory canal of the patient)	12	Not detected	Not detected	Not detected
**Total number of samples processed in this set: 15**	**Total number of patients: 8**	**Total microplastic count: 16 (Water control samples are not included)**	**Microplastic particle size range: 20–930 μm (Water control samples are not included)**	**Colors of the microplastics: black, transparent, blue, purple, dark blue**

## References

[1] Prata JC da Costa JP Lopes I Duarte AC Rocha-Santos T Environmental exposure to microplastics: An overview on possible human health effects Science of the Total Environment 2020 702 1 134455 10.1016/j.scitotenv.2019.134455 31733547

[2] Ragusa A Svelato A Santacroce C Catalano P Notarstefano V Plasticenta: First evidence of microplastics in human placenta Environment International 2021 146 106274 10.1016/j.envint.2020.106274 33395930

[3] Schmid C Cozzarini L Zambello E Microplastic’s story Marine Pollution Bulletin 2021 162 111820 10.1016/j.marpolbul.2020.111820 33203604

[4] Barceló D Picó Y Alfarhan AH Microplastics: Detection in human samples, cell line studies, and health impacts Environmental Toxicology and Pharmacology 2023 101 104204 10.1016/j.etap.2023.104204 37391049

[5] Karami A Golieskardi A Keong Choo C Larat V Galloway TS The presence of microplastics in com-mercial salts from different countries Scientific Reports 2017 7 46173 10.1038/srep46173 28383020 PMC5382780

[6] Kumar R Manna C Padha S Verma A Sharma P Micro(nano)plastics pollution and human health: How plastics can induce carcinogenesis to humans? Chemosphere 2022 298 134267 10.1016/j.chemosphere.2022.134267 35301996

[7] Wang YL Lee YH Hsu YH Chiu IJ Huang CC The Kidney-Related Effects of Polystyrene Microplastics on Human Kidney Proximal Tubular Epithelial Cells HK-2 and Male C57BL/6 Mice Environmental Health Perspectives 2021 129 5 57003 10.1289/EHP7612 33956507 PMC8101928

[8] Sun W Jin C Bai Y Ma R Deng Y Blood uptake and urine excretion of nano- and micro-plastics after a single exposure Science of The Total Environment 2022 848 157639 10.1016/j.scitotenv.2022.157639 35905964

[9] Zhang N Li YB He HR Zhang JF Ma GS You are what you eat: Microplastics in the feces of young men living in Beijing Science of The Total Environment 2021 767 144345 10.1016/j.scitotenv.2020.144345 33434834

[10] Wen S Zhao Y Wang M Yuan H Xu H Micro(nano)plastics in food system: potential health impacts on human intestinal system Critical Reviews in Food Science and Nutrition 2024 64 5 1429 1447 10.1080/10408398.2022.2116559 36066327

[11] Mssr T Pathak P Singh L Raj D Gupta DK A novel circular approach to analyze the challenges associated with micro-nano plastics and their sustainable remediation techniques Journal of Environmental Science and Health, Part A Toxic/Hazardous Substances and Environmental Engineering 2023 58 7 694 705 10.1080/10934529.2023.2208507 37150890

[12] Zhang P Liu Y Zhang L Xu M Gao L The interaction of micro/nano plastics and the environment: Effects of ecological corona on the toxicity to aquatic organisms Ecotoxicology and Environmental Safety 2022 243 113997 10.1016/j.ecoenv.2022.113997 35988380

[13] Hanege FM Kalcioglu MT Sargin F Cetinkaya Z Tekin M Does cerumen have a risk for transmission of HIV? European Journal of Clinical Microbiology & Infectious Diseases 2015 34 789 793 10.1007/s10096-014-2292-7 25480431

[14] Gholami-Parizad E Taherikalani M Mozaffar-Sabet NA Asmar M Gholami-Parizad S Cerumen as a potential risk for transmission of Hepatitis B virus Acta Microbiologica et Immunologica Hungarica 2011 58 2 105 112 10.1556/amicr.58.2011.2.3 21715280

[15] Bayindir Y Kalcioglu MT Durmaz R Ozturan O Detection of HCV-RNA in cerumen of chronically HCV-infected patients Laryngoscope 2005 115 3 508 511 10.1097/01.mlg.0000157828.00509.a0 15744167

[16] Liu S Lin G Liu X Yang R Wang H Detection of various microplastics in placentas, meconium, infant feces, breastmilk and infant formula: A pilot prospective study Science of The Total Environment 2023 854 158699 10.1016/j.scitotenv.2022.158699 36108868

